# Premature ovarian insufficiency in patients with thyroid-related poly-autoimmunity

**DOI:** 10.3389/fendo.2026.1907480

**Published:** 2026-09-07

**Authors:** Maria Flavia Bagaglini, Ilaria Stramazzo, Matteo Nocito, Poupak Fallahi, Alessandro Antonelli, Maria Giulia Santaguida, Lucilla Gargano, Francesca Ferrari, Enke Baldini, Salvatore Ulisse, Anna Carretti, Marco Centanni, Camilla Virili

**Affiliations:** 1 Department of Medico-Surgical Sciences and Biotechnologies, Sapienza University of Rome, Rome, Italy; 2 Azienda Sanitaria Locale (ASL) Latina, Latina, Italy; 3 Department of Translational Research and New Technologies in Medicine and Surgery, University of Pisa, Pisa, Italy; 4 Department of Surgical, Medical and Molecular Pathology and Critical Area, University of Pisa, Pisa, Italy; 5 Department of Surgery, Sapienza University of Rome, Rome, Italy

**Keywords:** Hashimoto’s thyroiditis, miscarriage, poly-autoimmunity, premature ovarian failure, premature ovarian insufficiency

## Abstract

**Background:**

An anticipated menopause is a disquieting health problem; its association with autoimmune disorders has been reported. However, data about the presence of premature ovarian insufficiency (POI) and early menopause (EM) framed in thyroid-related poly-autoimmunity are rather scanty. In this view, we described the presence of a precocious menopause in a large cohort of women with autoimmune thyroid disease (ATD) isolated or framed in poly-autoimmune syndromes.

**Study design:**

The characteristics of 7,690 postmenopausal women has been evaluated in a tertiary center for thyroid diseases. To be included, women must have been menopausal for at least 3 years without any menstrual bleeding. Three subgroups were examined and compared: women with nonautoimmune endocrinopathies (*n* = 5,697; group A), representing an internal control, or with isolated ATD (*n* = 1,689; group B) or with thyroid-related poly-autoimmunity (*n* = 304; group C).

**Results:**

In the whole sample, 213 (2.77%) women experienced unexplained precocious menopause, 115 (1.50%) showed spontaneous premature ovarian insufficiency (POI), and 98 (1.27%) experienced early menopause (EM). The prevalence of POI was higher in women with thyroid–related poly-autoimmunity (group C) than in group B and A (5.92 vs. 1.84 vs. 1.16%; *p* < 0.0001). A spontaneous miscarriage was observed in 52/213 women (24.4%) with precocious menopause (EM + POI), being significantly higher in women with poly-autoimmunity (3.61% vs. 0.53%, 0.71%) than those in groups A and B, respectively (*p* < 0.0001). Univariate analysis revealed that familiarity was the more relevant independent variable associated with POI in poly-autoimmunity.

**Conclusions:**

Our data strongly suggest that young women with thyroid-related poly-autoimmunity, born to a family with thyroid-related poly-autoimmunity, should have their ovarian reserve checked early to guide fertility preservation and family planning.

## Introduction

The number of oocytes in human ovaries progressively decreases in an age-related fashion during reproductive life until menopause (1). When a faster rate of oocyte depletion occurs, precocious menopause ensues. When menopause occurs before the age of 40, it is termed premature ovarian insufficiency (POI), and when it occurs between 40 and 45 years it is referred to as early menopause (EM) (1, 2). In the latest European Society of Endocrinology guidelines as well as in ESHRE’s guidelines (2, 3), a diagnosis of POI is extended to women with menstrual irregularity and those with serum FSH levels >25 IU/L before 40 years of age, with four or more months of secondary amenorrhea (3). POI may feature intermittent ovarian function, which was seen in a fraction of patients: spontaneous ovulation and, rarely, pregnancy may occur as well (1). However, the onset of POI may also occur without signs or symptoms, and the majority of women reported normal menarche age, normal menstrual cycle, and fertility (4), thus delaying the diagnosis of POI. Different pathogeneses have been described for POI—genetic, iatrogenic, autoimmune, etc.—but a large rate of unknown etiologies is reported (2, 3). An autoimmune origin of POI is usually hypothesized when anti-21-hydroxilase (anti-21OH) or anti-ovary autoantibodies are detected. However, the first, quite specific, is mostly associated with the fairly rare autoimmune Addison’s disease (5, 6), and the latter failed to meet the specificity and the sensitivity of a good marker (3). Following ESHRE’s guidelines, an autoimmune etiology for POI is considered probable when other autoimmune comorbidities are associated (3). Accordingly, it has been estimated that 10%–40% of women with POI have another autoimmune disease, most commonly autoimmune hypothyroidism (1). The role of autoimmunity and, specifically, ATD in the whole reproduction process spans over infertility, miscarriage, repeated pregnancy loss, POI (7–10), and thus many adverse pregnancy outcomes (7, 11, 12). So far, the association with other autoimmune disorders makes the presence of autoimmune POI highly probable (3, 5, 6), chiefly in the context of polyglandular autoimmune syndrome (PAS) types I and II, which are rare disorders (13). On the contrary, ATD is the most prevalent autoimmune disorder worldwide and plays a pivotal role in type III PAS (14), which encompasses both endocrine and nonendocrine autoimmune conditions (15, 16), including POI. The presence of POI in women with ATD-related poly-autoimmunity was never assessed, as these data are usually obtained by looking for ATD in women with POI (11, 12, 17). Therefore, we reversed the approach and we aimed at describing, in a large cohort of women, the features of POI occurring in ATD-related poly-autoimmune syndromes.

## Patients and methods

**Figure 1:**
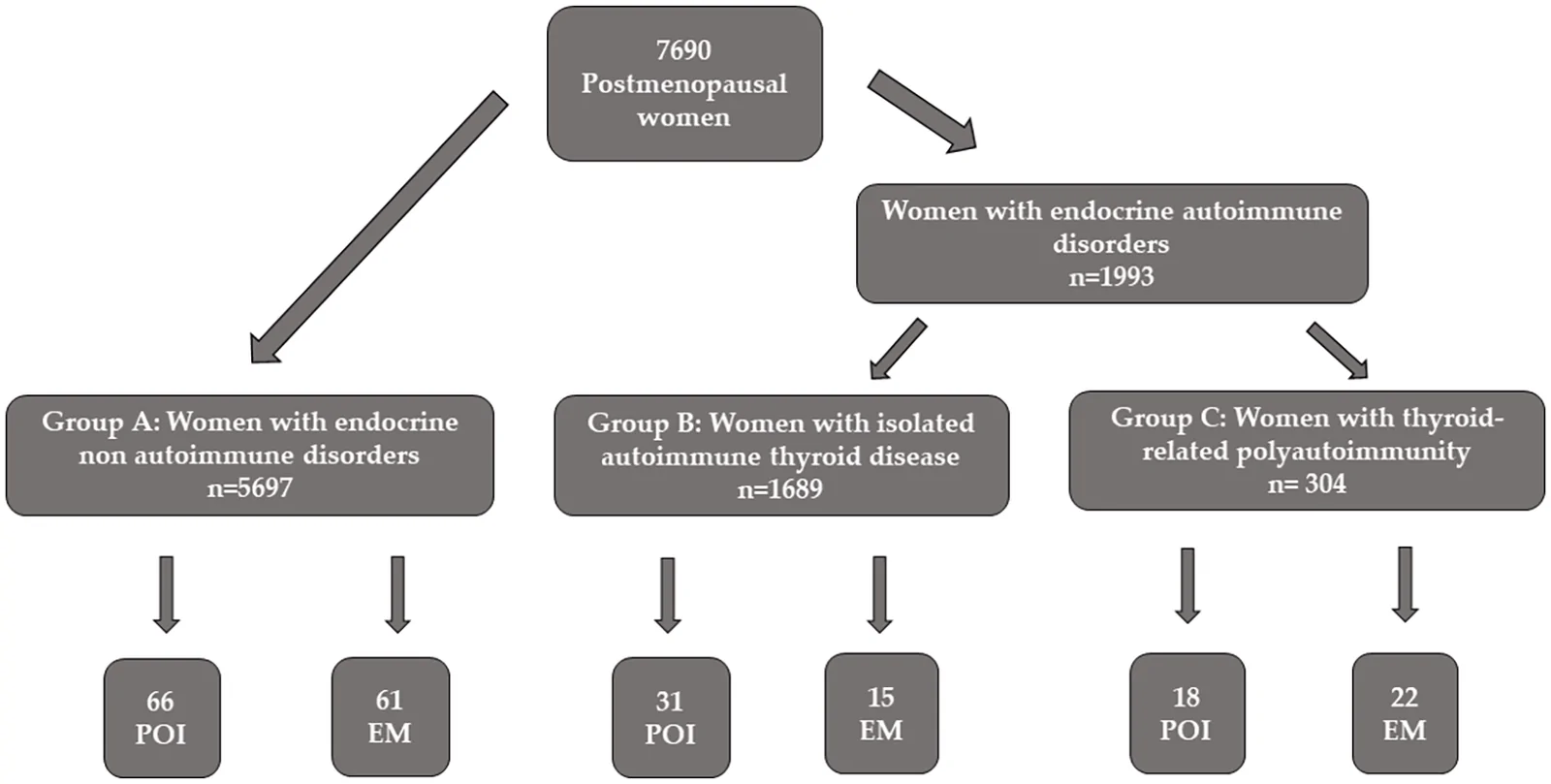
Schematic representation of postmenopausal women consecutively examined. Group A: women with endocrine disorders without autoimmunity, group B: women with isolated autoimmune thyroid disease, and group C: women with thyroid-related poly-autoimmunity. POI, premature ovarian insufficiency; EM, early menopause.

From the abovementioned cohort of women consecutively examined in a tertiary center for endocrine disorders at Sapienza University of Rome, Latina, we retrospectively analyzed data from 7,690 women who had definitely completed their fertile life for at least 3 years. These were subdivided into three groups: (A) 5,697 women with nonautoimmune endocrine disorders (used as internal control), (B) 1,689 women with isolated autoimmune thyroid disease, and (C) 304 women in which ATD was framed in poly-autoimmunity (**Figure 1**). Some anthropometric, biochemical and reproductive characteristics are shown **Table 1**. Poly-autoimmunity was defined as the occurrence of autoimmune thyroid disease as a pivotal disorder along with at least one more autoimmune disorder. Women with precocious menopause (POI + EM) were selected and evaluated for their clinical features and association with other autoimmune disorders. In this study, POI was defined as complete and irreversible secondary amenorrhea before the age of 40 and EM before the age of 45.

**Table 1:** Anthropometric characteristics of women with POI or EM.

Group of women	Type of menopause	Number	Familiar clustering^a^ (%)	BMI, median value (IQs)	Menarche, median value (IQs)	Menopause age, median value (IQs)	Parity (%)	Mother’s age at the time of first miscarriage, median value (IQs)	Serum TSH at first visit (mU/L)
Group A, nonautoimmune women	POI	66	13.6	27 (23–30)	12	39 (37–39)	85.4	28 (22–35)	1.34 (0.80–1.80)
	EM	61	19.7	27 (25–32)	12	43 (42–44)	84.9		1.76 (1.01–2.55)
Group B, autoimmune thyroiditis women	POI	31	25.8	25 (24–28)	12	39 (37–40)	96.8	30 (26–32)	1.80 (0.99–3.05)
	EM	15	33.3	27 (25–31)	12	43 (42–43)	93.0		2.40 (1.51–4.08)
Group C, poly-autoimmune women	POI	18	38.9	24 (22–29)	13	38 (35–39)	83.4	27 (25–30)	2.06 (1.34–3.21)
	EM	22	31.8	27 (26–30)	12	43 (42–45)	59.1		2.92 (1.87–5.17)

POI, premature ovarian insufficiency; EM, early menopause.

^a^POI + EM: A vs. B vs. C, *p* < 0.0296 (Fisher’s exact test); POI: A vs. B vs. C, *p* < 0.0477; EM: A vs. B vs. C, *p* = ns (Fisher’s exact test).

To be included, women must have been menopausal for at least 3 years without any menstrual bleeding. The exclusion criteria were (a) women ascertained genetic and/or iatrogenic (surgery, radiotherapy, and chemotherapy) or infectious etiology of POI, (b) women with POI whose mother or close relatives had POI, due to the known genetic-related fraction of POI pathogenesis and the lack of autoimmunity in these relatives, (c) women using estrogen–progestin and any other interfering treatment before precocious menopause occurred, and (d) women with ascertained absence of concomitant disorders causing oligo-amenorrhea (PCOs, adrenogenital syndromes, hyperprolactinemia, etc.). Information was obtained at first visit, as ascertained by their record or by directly asking the women about menarche, miscarriage (age of the mother and trimester when it occurred), menopause and familial heritage, etc. To minimize possible mistakes in self-reported data, this information was checked again and verified during the next medical examinations. Kinship familiarity for autoimmune disorders was defined as the presence of autoimmune disorders (any kind) restricted to the mother, father, grandfather, grandmother, brothers, sisters, daughters, and sons.

### Diagnostic workup

The diagnosis of ATD and each autoimmune disorder was based on clinical, serological, and histological examinations. Indeed the diagnosis of HT was based on the presence of at least two of these three criteria: characteristic ultrasonographic pattern and repeated high titers of anti-thyroperoxidase antibodies (antiTPOAbs) and/or hypothyroidism, as previously described (18). Graves’ disease has been diagnosed on clinical ground and confirmed by the presence anti-TSH receptor antibodies and biochemical hyperthyroidism. The diagnosis of autoimmune atrophic gastritis (AAG) was suspected on the basis of unexplained anemia and the presence of anti-parietal cell antibodies, which was always confirmed by histological examination of multiple specimens obtained at gastroscopy, as described previously (19). Atrophy of the gastric mucosa was defined as focal or complete loss of parietal cells and/or replacement by metaplastic, pyloric, or intestinal glands. The degree of gastritis was assessed according to the updated Sydney System and its relative score (19, 20). Celiac disease had been diagnosed by the presence of positive anti-endomysium (EMA) and anti-transglutaminase (tTG) antibodies and confirmed by endoscopy. Their histological damage has been classified according to the modified Marsh Classification (21). The diagnosis of psoriasis was based on the presence of plaque psoriasis, a symmetric, erythematous lesion with silvery scales. The plaques were mostly located on the scalp, trunk, buttocks, and extremities. The uncertain appearance of the disease has been histologically confirmed (22). The diagnosis of psoriatic arthritis was based on the clinical identification of characteristic features (arthritis and/or spondylitis and/or enthesitis and/or dactylitis) in a patient with either active or clinically remitted cutaneous and/or nail psoriasis. The diagnosis was further supported by evidence of elevated inflammatory markers and by radiographic findings of ill-defined juxta-articular new bone formation in the absence of osteophytes in the affected joints (23). The diagnosis of rheumatoid arthritis was based on family history, clinical examination, joint evaluation through ultrasound, and laboratory markers such as serum C-reactive protein (CRP) and erythrocyte sedimentation rate (ESR) as well as anti-citrullinated protein antibodies (24). The diagnosis of ulcerative colitis was made based on clinical signs and symptoms, such as abdominal pain and bloody diarrhea, and recto-colon-sigmoidoscopy with biopsy. Histological examination revealed cryptitis, crypt abscesses, architectural distortion of the crypts, basal lymphocytosis, and distal Paneth cell metaplasia (25).

The diagnosis of each systemic autoimmune disease associated with ATD was confirmed by a proper expert specialist, according to the criteria approved by specific guidelines or consensus conferences (26–28). The presence of anticipated menopause in women with autoimmune disorders associated to thyroid autoimmunity in the frame of polyautoimmunity is reported in **Table 2**.

**Table 2:** Associated autoimmune diseases in patients with thyroid-related poly-autoimmunity and anticipated menopause.

POI	*n*	EM	*n*	POI + EM	AITD + autoimmune disorder, *n*	% of disease frequency
Chronic atrophic gastritis	7	Chronic atrophic gastritis	7	14	14/104	13.5
Psoriatic arthritis	1	Psoriatic arthritis	4	5	5/20	25.0
Psoriasis	3	Psoriasis	3	6	6/92	6.5
Vitiligo	1	Vitiligo	4	5	5/93	5.4
Sjögren’s syndrome	3	Sjögren’s syndrome	1	4	4/16	25.0
Antiphospholipid Abs syndrome/SLE	1	Antiphospholipid Abs syndrome/SLE	2	3	3/31	9.7
Rheumatoid arthritis	1	Rheumatoid arthritis	3	4	4/42	9.5
Undifferentiated connective tissue disease	2			2	2/20	10.0
Celiac disease	1			1	1/67	1.5
Type 1 diabetes mellitus	1			1	1/19	5.3
Addison’s disease	1			1	1/3	33.3
Autoimmune thrombocytopenia	1			1	1/11	8.3
Ulcerative colitis	1			1	1/23	4.3
		Multiple sclerosis	1	1	1/32	3.1

In the last two columns on the right, the absolute numbers were sized with the effective numbers of the single diseases associated to autoimmune thyroiditis.

POI, premature ovarian insufficiency; EM, early menopause; SLE, systemic lupus erythematosus.

### Ethical statement

Formal approval by the ethical committee was not required in accordance with the Italian regulation for non-interventional (observational) retrospective studies concerning human beings (AIFA Guidelines for Observational Studies, see https://servizionline.aifa.gov.it). However, a written informed consent was obtained from the women with POI. All personal data were stored in a tightly controlled repository and on a PC with a changing password.

### Statistical analysis

Statistical analysis was performed using the statistical software package IBM SPSS for Windows, V.27 (IBM Corp., Armonk, NY, USA). Median (interquartile range [IQ]) has been used to describe continuous variables analyzed by non-parametric Mann–Whitney *U*-test. Kruskal–Wallis non-parametric test has been used to analyze the medians of more than two groups. Pearson’s chi-square test was applied to the different categories of patients with and without POI. When the contingency table had more than 20% of cells with an expected count of less than five and the total number of cases was high, Fisher’s exact test was conducted using Monte Carlo simulation with 10,000 iterations.

## Results

**Figure 2:**
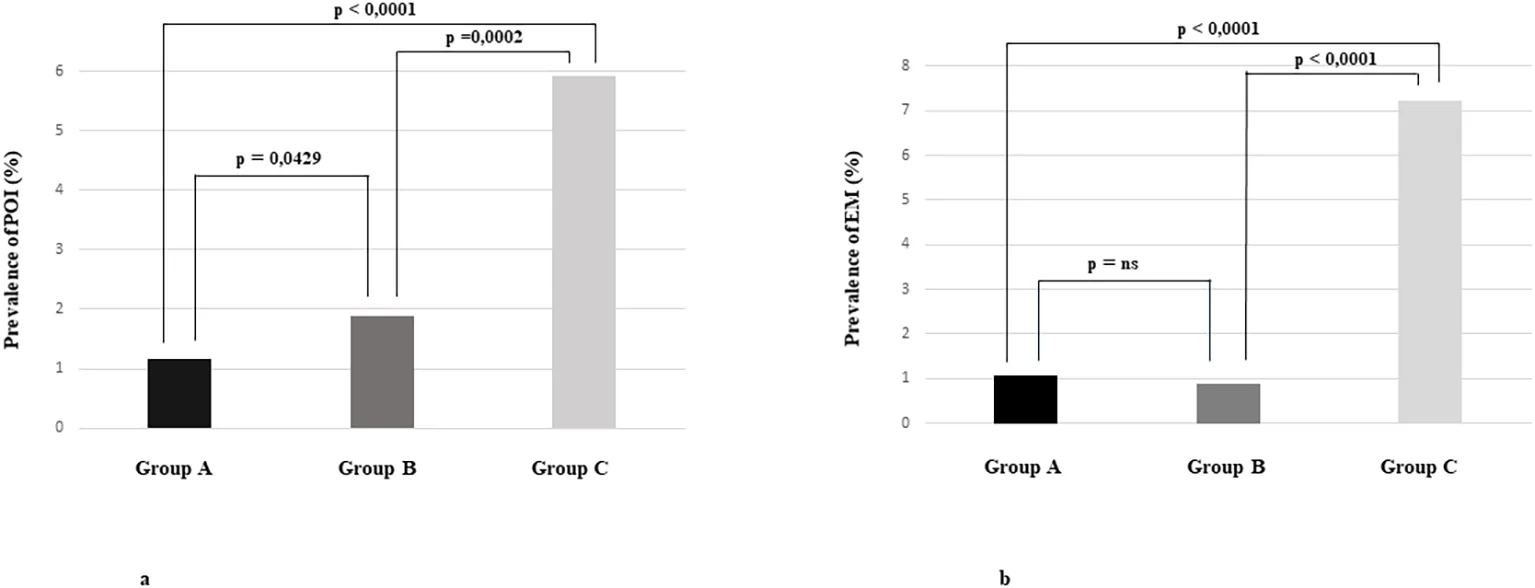
Prevalence of POI **(A)** and EM **(B)** in the three groups of patients. Group A: women with endocrine disorders without autoimmunity, group B: women with isolated autoimmune thyroid disease, and group C: women with thyroid-related poly-autoimmunity. POI, premature ovarian insufficiency; EM, early menopause.

From a large database encompassing approximately 20,000 endocrine patients, we recruited 7,690 women consecutively examined who had definitely completed their fertile life for at least 3 years. A total of 226 women (2.94%) had early non-iatrogenic menopause or premature ovarian failure, but 13 of these women were excluded because they had mothers or other first-degree relatives with POI, suggesting a genetic pathogenesis of the failure. Of these, five women had no autoimmune thyroid disorders and eight had autoimmune disorders (four with isolated ATD and four with poly-autoimmunity). None of them reported first-degree relatives with concurrent autoimmunity. Among the remaining 213, we identified 115 women (1.51% of the whole sample) who showed POI and 98 (1.27% of the whole sample) with EM, whose characteristics are shown in **Table 1**. Only one woman with POI had Graves’s disease, while the remaining cases were diagnosed as Hashimoto’s thyroiditis. At first, these women were subdivided in two groups: women with nonautoimmune endocrinopathies and women with autoimmune thyroid disorders. A precocious menopause (POI + EM) affected 2.23% of the women in the first group and 4.32% in the second group (*p* < 0.0001; OR = 1.978). Similarly, the prevalence is more than doubled when the analysis was restricted to women with POI: 1.16% in the nonautoimmune group and 2.46% in the autoimmune group (*p* < 0.0001; OR = 2.151). We further subdivided the women with autoimmune disorders as those with isolated autoimmune thyroid disorders (group B) or those with thyroid-related poly-autoimmunity (group C) (**Figure 1**). The median values of TSH at first visit, although slightly different (1.40 vs. 2.10 vs. 2.72 mU/L), were in the normal range. The kinship familial heritage for autoimmune disorders in women with precocious menopause was significantly different (*p* = 0.0296) among the three groups, which was higher in women with poly-autoimmunity (**Table 1**). Univariate analysis revealed that familiarity was the more relevant independent variable associated with POI in poly-autoimmunity. The prevalence of POI and EM in women with autoimmune disorders has been compared among the three groups. It has been observed that 31 out of 1,689 women (1.84%) in group B and 18 out of 304 women (5.92%) in group C (B vs. C: *p* = 0.0002; OR = 3.366) had POI, compared with only 1.16% observed in group A (A vs. B: OR = 1.545; chi-square test: *p* < 0.0001) (see **Figure 2A**). Similarly, EM has been observed in 1.07% in group A and 0.89% in group B (*p* = 0.0429), while in women with poly-autoimmunity it was much higher (OR = 7.23%; chi-square test: *p* < 0.0001) (see **Figure 2B**).

The duration of fertile life was obviously reduced in these women with POI. A median of 26 years of fertile life has been observed in groups A and B and 25 years in the poly-autoimmunity group compared with the usual duration of 39 to 40 years. Menarche occurred at a median age of 12 in both groups A and B, while in the poly-autoimmunity group it occurred at age of 13, but the difference did not reach statistical significance (**Table 1**). Parity in women with POI was comparable to what was described by other authors: it was almost two children per woman, and this was similar (83 vs. 96 vs. 89%; X square: *p* = ns) across all groups. At the time of menopause, the women were all euthyroid in group A, while 28 women were euthyroid (five already treated with thyroxine) and three were subclinical hypothyroid in group B and 17 were euthyroid (three already treated with thyroxine) and one was subclinical hypothyroid in group C.

**Figure 3:**
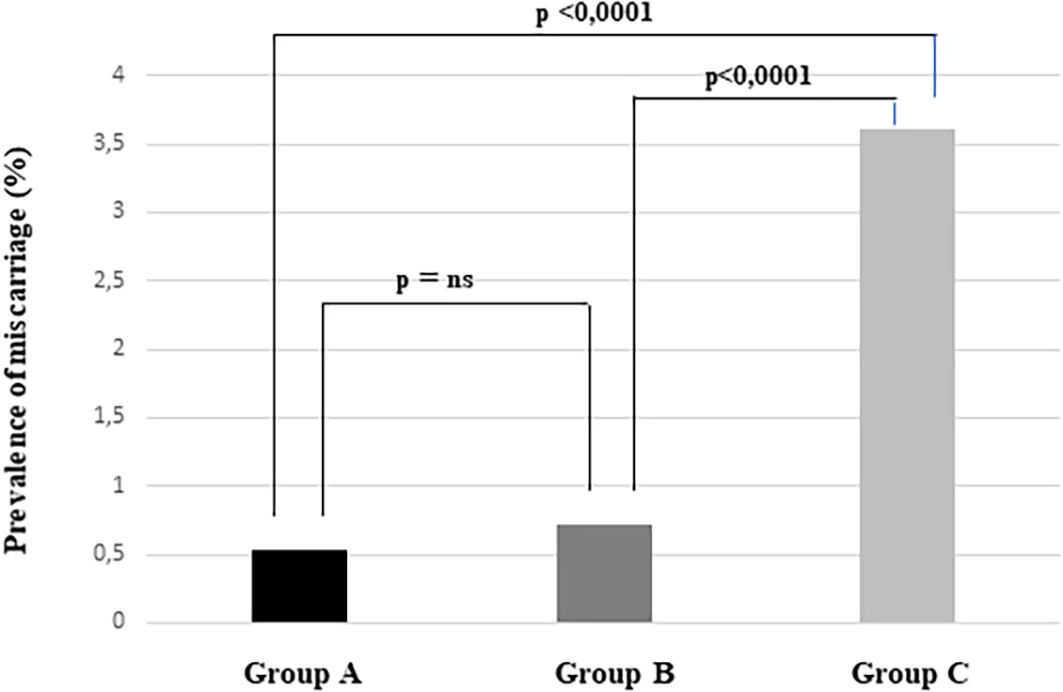
Prevalence of spontaneous miscarriage in the first trimester in women with anticipated menopause (POI + EM). Group A: women with endocrine disorders without autoimmunity, group B: women with isolated autoimmune thyroid disease, and group C: women with thyroid-related poly-autoimmunity. POI, premature ovarian insufficiency; EM, early menopause.

**Figure 2** shows the prevalence of spontaneous miscarriage in the first trimester of pregnancy in the whole sample. Among the 213 women with precocious menopause (EM + POI), a total of 52 women (24.4%) had at least one miscarriage during the first trimester of pregnancy. Upon analyzing the whole sample of each group, a miscarriage during the first trimester of pregnancy occurred in 0.53%, 0.71%, and 3.61% in groups A, B, and C, respectively (**Figure 3**). Focusing the analysis on the women with POI, this difference was maintained, being highly significant among the three groups and far prevalent in poly-autoimmune women (A = 0.37%, B = 0.53%, and C = 1.64%; *p* = 0.0048). The percentage of miscarriage was higher in women with POI than the one observed in the EM group (30.2% vs. 17.7%; *p* = 0.038). Only three women presented subclinical hypothyroidism at the first visit, and no relationship was observed between the serum TSH at first visit and the occurrence of miscarriages. However, we only had data to confirm that 35 out 52 women were euthyroid at the time of miscarriage, although they were equally distributed among the groups. Age was not a confounding factor as no miscarriage occurred after the age of 38, and there was no difference among the three groups (**Table 1**).

We further analyzed the more frequent autoimmune disorders associated with precocious menopause (POI + EM) in the frame of poly-autoimmunity. In addition to thyroid autoimmunity, 12/18 women had only one comorbidity, while six out 18 (33%) had multiple associated autoimmune disorders, an event that is less frequent in women with EM (3/22; 13.6%). Among the autoimmune disorders, atrophic gastritis was the most frequently observed autoimmune comorbidity, followed by rheumatic disorders and cutaneous autoimmunity (see **Table 2**). However, when the number of anticipated menopauses was sized against the frequency of association of these disorders, psoriatic arthritis was the more and celiac disease the lesser associated to POI and EM. Indeed Fisher’s exact test upon Monte Carlo simulation revealed a significantly different distribution of precocious menopause (POI + EM) (*p* = 0.019).

## Discussion

Precocious menopause is a disquieting problem owing to the loss of the ability to conceive as well as to the long-term consequences of premature reduction of estrogen signal in different tissues, including adverse effects on cognition, mood, and cardiovascular, bone, and sexual health as well as an increased risk of early mortality (29, 30),. Despite the fact that in a large number of cases the etiology of POI remains uncertain (3, 5, 11, 14), it has been estimated that autoimmune etiology covers 4%–40% of all POI cases (7, 14). POI may occur associated with Addison’s disease (APS 2): some studies reported 10%–20% of coexistence of these disorders (5, 6, 11), while the occurrence of autoimmune thyroid disease (ATD) has been reported in 33.7% of patients with POI (9, 11, 12, 14, 31). These high rates of association were derived from studies carried out in cohorts of patients with POI, looking for the association with further autoimmune disorders. We reversed the point of view instead and compared the prevalence of POI in a large cohort of women with ATD isolated or framed in poly-autoimmunity, with autoimmune thyroid disorders being the far more prevalent ones in fertile women. In our study, it appears that the prevalence of POI in the setting of thyroid-related poly-autoimmunity is five times higher than in patients without autoimmune disorders and three times more than in women with isolated ATD. It is worth noting that this prevalence is significantly higher than 1% of premature ovarian failure and even than 1%–3% as more recently reported using more relaxed criteria (2, 3, 30) in the general population. Indeed the recent and more extensive definition of POI (3) encompasses all pathogeneses of this disorder and even the reversible forms (2, 3). Therefore, even thyroid-related poly-autoimmunity seems to cluster fertile women at risk of POI. In this context, chronic atrophic gastritis, the most common ATD-related organ-specific disorder (15–17), was the more frequent disease associated with POI. However, once normalized for the frequency of ATD-associated disorders, the contingency table revealed psoriatic arthritis as having the relatively more common association with POI. The link between psoriatic diseases and reproductive life has been described (32), but its pathogenesis remains to be elucidated. Overall, we have no evidence that a specific ATD-associated disorder may be responsible for an increased prevalence of POI in poly-autoimmunity. On the contrary, the spectrum of both organ-specific or systemic-associated autoimmunity disorders suggests that the imbalance of cellular immunity, a common flag to these multiple autoimmune disorders, may be a significant cause of precocious menopause as described in (33) (**Table 2**). We were not surprised not to find a significant association of POI with Addison’s disease as this disease is strongly associated with POI but is approximately 300 times less frequent than ATD and was therefore used as a pivotal autoimmune disease. The presence of Addison’s disease remains the more specific diagnostic tool to detect poly-autoimmunity and even POI, but its rarity pushed us to prefer the presence of ATD as “truffle dog” to detect a larger number of autoimmune disorders otherwise occult or unrelated each other. The possibility that some autoimmune disorder may cluster preferentially with another is more than a possibility (see type 1 diabetes and celiac disease) and represents the evolution of associative studies.

In most studies, it was reported that POI was related to hypothyroidism (9, 11, 12). The most important etiology of nonsurgical hypothyroidism in adults is ATD (34). In our sample, a role for thyroid failure, if any, appears to be residual as only four patients out of 49 were subclinical hypothyroid at the time of POI onset. This suggests that the impairment of ovarian function appears to be related to immunologic derangement rather than to reduced thyroid function, although this may follow the appearance of ATD. Besides the role of genetic factors described by Federici et al. (35), the present data strongly support that immunological mechanisms may play a pivotal role in determining POI, in keeping with those published by Savukoski SM et al. (36).

The analysis of the miscarriage in the first trimester of pregnancy, in women either with precocious menopause (EM + POI) or with POI alone, revealed that its rate was significantly higher in poly-autoimmune than both in isolated HT women and in those with nonautoimmune endocrinopathies. Furthermore, the overall miscarriage rate in poly-autoimmune patients was three times higher than that reported in the general population (37).

Some papers demonstrated that higher TSH levels in women correlated with the risk of miscarriage and repeated pregnancy loss (see, for review, [38]). However, the authors themselves acknowledged that there was a large variability in the timing of TSH measurements and that TPO antibodies were not routinely assessed. Thus, whether hypothyroidism itself or the presence of autoimmunity might determine miscarriage is as yet an open question. In our study, serum TSH levels, at first visit, positively correlated with the presence of autoimmunity, but not with the rate of miscarriage. This suggests that the increased occurrence of miscarriage in these women would be more consistently associated to the deeper derangement of autoimmune context. These results are in keeping with those reported above in a systematic review (38), which suggested: “some other systematic clinical condition(s) independently affects ovarian reserve and miscarriage risk”. Auto-aggressive processes, often occult or disregarded, may represent the missing link as shown for repeated pregnancy loss (8).

The main limitation of our study stems from its retrospective design. However, the large sample even tested for autoimmune heritage, the rigorous enrollment using thyroid autoimmune diseases as pivotal disorder, and the definite diagnosis of associated autoimmune disorders limited this weakness and allowed us to describe the impact of thyroid-related poly-autoimmunity on POI and early menopause. We acknowledge that the presence of Addison’s disease may, in this particular setting, be more specific in addressing a POI diagnostic workup. However, the far higher prevalence of thyroid autoimmunity and its earlier occurrence (13–16), compared with that of Addison’s disease, grant better sensitivity in the early detection of POI. Overall, our present data suggest that young women with thyroid-related poly-autoimmunity and born to a family with thyroid autoimmunity and/or poly-autoimmunity are at risk of POI and should have their ovarian reserve checked early to guide fertility preservation and family planning.

## Data Availability

The raw data supporting the conclusions of this article will be made available by the authors, without undue reservation.
